# Alignment of color discrimination in humans and image segmentation networks

**DOI:** 10.3389/fpsyg.2024.1415958

**Published:** 2024-10-23

**Authors:** Pablo Hernández-Cámara, Paula Daudén-Oliver, Valero Laparra, Jesús Malo

**Affiliations:** Image Processing Lab, Parc Científic, Universitat de València, València, Spain

**Keywords:** vision models, color discrimination, image segmentation, artificial neural networks, U-Nets, image statistics, chromatic adaptation, Divisive Normalization

## Abstract

The experiments allowed by current machine learning models imply a revival of the debate on the causes of specific trends of human visual psychophysics. Machine learning facilitates the exploration of the effect of specific visual goals (such as image segmentation) by different neural architectures in different statistical environments in an unprecedented manner. In this way, (1) the principles behind psychophysical facts such as the non-Euclidean nature of human color discrimination and (2) the emergence of human-like behaviour in artificial systems can be explored under a new light. In this work, we show for the first time that the *tolerance* or *invariance* of image segmentation networks for natural images under changes of illuminant in the color space (a sort of insensitivity region around the *white*) is an *ellipsoid* oriented similarly to a (human) MacAdam ellipse. This striking similarity between an artificial system and human vision motivates a set of experiments checking the relevance of the statistical environment on the emergence of such insensitivity regions. Results suggest, that in this case, the statistics of the environment may be more relevant than the architecture selected to perform the image segmentation.

## 1 Introduction

**Natural images and principled explanations in vision science**. A long-standing hypothesis in vision science assumes that sensory behaviour derives from an evolutionary adaptation to the regularities of the environment (Barlow, 1959, 2001). This hypothesis is statistical in spirit because it assumes that certain architecture (network of sensors and neurons) is progressively updated to become optimal according to certain task for the inputs faced by the system (Richards et al., 2019). As a result, the concept of *natural images* has become central in this kind of principled explanation (Field, 1987; Simoncelli and Olshausen, 2001; Torralba and Oliva, 2003; Hyvärinen et al., 2009), because it refers to stimuli (e.g., photographic images) which are representative of certain visual environments and constitute the training set for the system.

**Linear statistical models in color vision**. The link between color vision and the statistics of the natural environment has a long and fruitful history. Classical approaches often employ linear models to explain different aspects of color vision. For instance, one seminal study derived opponent color channels from the statistics of color samples (Buchsbaum and Gottschalk, 1983): authors assumed that the goal of the color sensors is to decorrelate the neural responses after the photoreceptors so they computed the linear Principal Component Analysis (PCA) of color samples in natural images. PCA transforms data into a set of linearly uncorrelated components (Jolliffe, 2002), identifying the directions (principal components) in which the data varies the most. It turns out that the best directions to encode natural colors are the *luminance*, the *red-green*, and the *yellow-blue* directions. This statistical explanation of the physiological achromatic and opponent channels (Shapley and Hawken, 2011) is more conclusive than classical hue cancellation experiments (Vila-Tomás et al., 2023). This is because the opponent spectral sensitivities obtained from PCA are more similar to the final sensitivities in multi-stage models such as (DeValois and DeValois, 1993), while hue cancellation results are mainly determined by the experimental choice of the cancellation stimuli (Vila-Tomás et al., 2023). Another notable linear approach involved the derivation of chromatic Contrast Sensitivity Functions (CSFs) through linear filters designed to maximize information transmission (Atick and Redlich, 1992; Atick et al., 1992). In this case the authors also assumed a decorrelation goal but in the presence of retinal noise. The optimal filters amplify certain spatial frequencies to whiten the responses (to make their spectrum flat) while attenuating the spatial frequencies where the noise is bigger than the typical signal. Additionally, explanations of chromatic adaptation, a process by which the visual system adjusts to changes in the lighting conditions, have been based on linear shifts in the average and covariance of color samples (Webster and Mollon, 1997; Clifford et al., 2007). The average represents the mean color value, while the covariance indicates how color values vary together, providing insights into the overall color distribution in the visual scene. Adaptation is understood as a transform to an invariant inner representation that compensates for the color shifts induced by changes in the environment (illumination, shadows, etc.). In summary, linear statistical models have identified opponent chromatic channels, the frequency bandwidth for achromatic and opponent chromatic patterns, and adaptation mechanisms based on the mean and the covariance of the chromatic signals.

**Nonlinear statistical models in color vision**. More recently, nonlinear descriptions of color statistics have been used to reproduce the nonuniform resolution and adaptation of the response of opponent mechanisms. In particular von der Twer and MacLeod (2001); MacLeod and von der Twer (2003) suggested that the nonlinear behaviour of opponent channels could be explained by using univariate Cumulative Density Functions (CDFs) of color samples. The CDF transforms the input probability into a uniform probability. This means that if the sensor responses are related to the CDF, simple uniform resolution in the response domain minimizes the error introduced in the representation of the signal (Lloyd, 1982). This philosophy was further extended to other optimization principles and higher-dimensional scenarios using Sequential Principal Curves Analysis (SPCA), a statistical method that generalizes PCA by fitting smooth curves through the data allowing for the representation of nonlinear structures. The different nonlinearities that can be accommodated in SPCA (Laparra and Malo, 2016) extend the cumulative density approach from optimal error minimization (Lloyd, 1982) to optimal information maximization (Laughlin, 1983). In this way, new explanations of color adaptation, color constancy and color illusions were proposed (Laparra et al., 2012; Laparra and Malo, 2015).

**Signal statistics and model architecture**. By definition, nonlinear models are more accurate and general than linear models. However, the above nonlinear descriptions of color phenomena were more focused on the statistics of the color signals rather than on the architecture, i.e., they oversaw the specific network required for the implementation of the computations. In general, the interactions between the statistical goal and architecture are not trivial (Poggio, 2021; Hernández-Cámara et al., 2023a; Hernández-Cámara et al., 2024). For example, different deep-learning architectures trained according to the same statistical goal may lead to critically different behaviours. This has been the case in studying color illusions (Gomez-Villa et al., 2020), or chromatic contrast sensitivity, either from low-level (Li et al., 2022), or higher-level principles (Akbarinia et al., 2023). In these studies authors show that *for the same functional goal* deeper networks may get better performance in the goal, but they display less-human behaviour than shallow networks (in terms of bandwidth or visual illusions).

**Open issues in statistical explanations of color discrimination**. The metric of the tristimulus space is not Euclidean, for instance, the discrimination region around the white has a specific asymmetry and orientation (MacAdam, 1942). Current statistical explanations of that fact are based on very low-level principles: error-minimization or information-maximization using SPCA (Laparra et al., 2012) or Gaussianization techniques (Jiménez et al., 2013), or the techniques based on Fisher information (da Fonseca and Samengo, 2016, 2018) which is another form of information maximization. Neither of these explanations take the architecture of the system into account (they only describe the properties of color distributions), and the principles are so low-level that are not directly connected to actual visual tasks.

**Questions addressed in this work:**

- *Is it possible to derive basic properties of human color discrimination ellipses from visual tasks of higher-level than error-minimization or information-maximization?* Particularly [as opposed to the cited low-level literature (Laparra et al., 2012; Jiménez et al., 2013; da Fonseca and Samengo, 2016, 2018)] by explicitly optimizing a neural architecture with certain resemblances to the retina-cortex pathway.
- In solving the considered higher-level visual task, *what is the relative relevance of the color statistics of the environment versus the consideration of reasonable variants in the network architecture?*

In this work, we address these questions using networks trained to perform image semantic segmentation (Guo et al., 2018), which is a mid-level vision task that consists of identifying the objects in the input images by classifying each pixel into one semantic category. We implement this task using variants of the successful U-net architecture (Ronneberger et al., 2015). The encoding part of this architecture is a cascade of linear-nonlinear stages which displays certain resemblances (in connectivity and function) with early vision (Jacob et al., 2021). Moreover, we augment the conventional U-net by including biologically-inspired layers, the so-called *Divisive Normalziation* (DN) (Hernández-Cámara et al., 2023b). This DN layer is a canonical non-linearity in sensory neuroscience (Carandini and Heeger, 2012) that takes into account the inhibitory effect of neighbour neurons and explains chromatic adaptation too (Abrams et al., 2007; Hillis and Brainard, 2005). Finally, to check the relevance of the statistics of the environment, we conduct the training and testing of the networks with different kinds of images with distinctly different color statistics.

The idea is to check if human-like tolerance regions to color changes emerge in these networks tuned to solve semantic image segmentation. And, if they sometimes do, does it depend more on the statistics of the environment or on the variants introduced in the architecture?

In this work, we report the following finding: the region of invariance to changes in illumination in image segmentation networks trained with naturally illuminated images is similar to the region of insensitivity (or invariance) to color changes in humans (the MacAdam ellipse around the white). Therefore, this mid-level task may be an alternative to previous lower-level explanations. However, we find that the statistics of the colors in the environment are more relevant to explain color discrimination than the considered variants in the architecture in the segmentation network.

## 2 Materials and methods

Here we introduce the six methodological elements required for our experiments: (1) various distinct chromatic environments for the segmentation goal: a naturally illuminated scenario (regular photographic scenes with daylight illumination), and then two counter-examples selected to have quite different color statistics (submarine images and achromatic images respectively). Then, (2) we outline the methodology we follow to compare the tolerance to color changes in artificial networks and in humans. As this general methodology implies generating consistent color shifts in scenes annotated for segmentation, (3) we select one of the possible approximated ways to introduce such color shifts, namely the variation of a simulated spectral illumination. Then, (4) we illustrate the shape of the tolerance of humans to color shifts around the white color (or the anisotropy of that MacAdam ellipse), (5) we present the scenes with shifted colors to check the tolerance of the networks, and finally, (6) we present details of the neural architectures of the considered image segmentation networks.

### 2.1 Environments of different statistics

**Figure 1:**
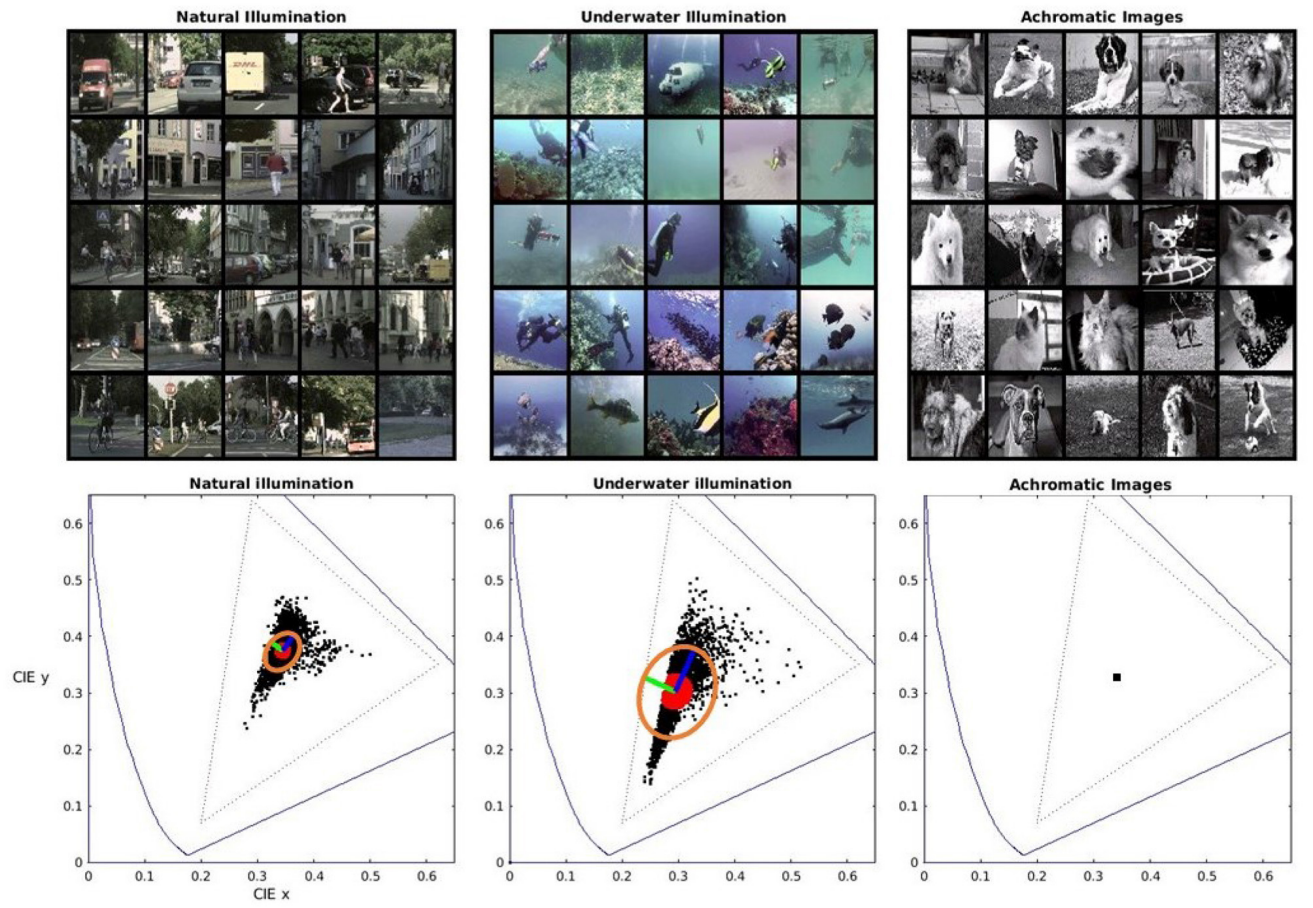
Different environments **(top)** and associated color statistics **(bottom)**. *Left:* daylight natural illumination. *Center:* daylight+underwater filtering and scattering. *Right:* artificial achromatic scenes (flat spectral reflectances and equienergetic illuminant). The corresponding 1931 CIE xy diagrams show representative color samples from the scenes (in black) and the closest neighbours to the average chromaticity (in red). It also shows local principal components (in green and blue) and the associated ellipsoid (in orange) computed from the local Principal Component Analysis (local PCA) of the nearest neighbours to the average chromaticity.

The analysis of color discrimination of different image segmentation networks requires training these artificial systems in visual environments with substantially different color statistics. The idea is checking if differences in color statistics induce consistent changes in color discrimination. To this end, we considered three datasets with known segmentation ground truth, but distinct scene statistics: *Cityscapes* (Cordts et al., 2016),^1^ *SUIM* (Islam et al., 2020),^2^ and *Oxford-IIIT Pets* (Parkhi et al., 2012).^3^ While *Cityscapes* consists of a range of urban photographic scenes under natural illumination (see Figure 1), the other two environments are counter-examples specifically selected to have distinct color statistics. On the one hand, *SUIM* is shifted to blue because it consists of underwater pictures. On the other hand, while *Pets* also has natural daylight illumination, we intentionally removed all chromatic information by changing the images to gray-scale so that the segmentation has to be based on alternative (non-chromatic) visual features such as shape or texture. The term “natural scenes” is applicable to the “urban scenes” in CityScapes, because “natural” refers more to the low-level statistical features of the images (smoothness, edge consistency and continuity, or day-light illumination) rather than to the presence of natural versus man-made objects. In fact, similarly to the achromatic literature (Field, 1987; Olshausen and Field, 1996), when dealing with spatio-chromatic scenes Gabor-like sensors in chromatically-opponent channels emerge both in forest-landscape scenes and in urban scenes (Doi et al., 2003; Gutmann et al., 2014).

The color statistics of these environments are illustrated by the scatter plots of color samples in the 1931 CIE xy chromatic diagrams in Figure 1. In these diagrams, the spectral locus and the triangle defined by the red, green and blue primaries of regular displays have been plotted for useful reference. For each environment, we took the 1000 nearest neighbours to the average chromaticity and computed the local Principal Component Analysis (local PCA) as in Laparra et al. (2012) and Laparra and Malo (2015). The local principal components (in green and blue) and ellipses (in orange) associated with the local covariance matrices from the local PCA highlight the difference in color statistics. Therefore, systems trained for information maximization or error minimization in these environments should have different metrics when considering color differences. Of course, nothing can be said for systems trained in (artificially) achromatic environments.

A technical note on the color of the databases. The images in the considered databases are expressed in digital values. This device-dependent color representation is transformed into standard 1931 CIE XYZ tristimulus vectors assuming a standard display calibration (Hunt, 2005; Malo and Luque, 2002). To simplify the implementation of the experiments involving changes of illumination in the following sections, we reduced by a factor 0.75 the excitation purity of all the colors in *Cityscapes* and *SUIM*. This can be easily seen in the sharp edge in the cyan colors of the underwater environment. Incidentally, this sharp edge suggests that camera recordings in this region are already saturated in the blue channel. This bias does not represent a problem for our study because this is just a counter-example with substantially non-natural statistics. We applied this small reduction because changes in spectral illumination imply movements of the color manifold towards the limits of the color gamut that can be properly represented in digital systems (the triangle in dotted style). This reduction in the original saturation allows stronger changes in the illumination. Nevertheless, it is important to note that this does not change the relative shape of the color distributions (does not change the orientation of the covariance matrices nor its relative size) and then, it does not modify the generality of the results.

### 2.2 Comparing tolerance to color shifts in humans and in machines

Color discrimination in humans has been defined in different ways depending on the stimuli and experimental task done by the observers. For instance, the classical MacAdam results are based on the variability of color matching experiments with flat patches of light sources (MacAdam, 1942; Wyszecki and Stiles, 2000). The covariance of this variability leads to the well-known ellipses in the 1931 CIE xy diagram. However, detection thresholds of deviations in different chromatic directions using randomly textured stimuli (Barbur, 2004) leads to ellipses with the same shape and orientation but larger size, about a × 5 factor in size (Jennings and Barbur, 2010). Similar detection thresholds measured with natural images under controlled changes in illumination (Alabau-Bosque et al., 2024) are compatible with the results by Jennings and Barbur (2010).

All these descriptions are qualitatively equivalent: the relevant facts (Stockman and Brainard, 2010; Wyszecki and Stiles, 2000) that should be reproduced by the models is that the higher sensitivity (lower threshold and equivalently lower variability of the color matches) is observed in a specific *red-green* direction while the lower sensitivity (bigger threshold and bigger variability) is in an almost orthogonal *yellow-blue* direction. In Section 2.5 we visually illustrate that these are really robust trends.

In the case of artificial networks we will use the concept of *tolerance region*. Note that the performance of the neural net in the visual task (in this case segmentation) has a certain value given that the images are illuminated as in the training conditions (and hence the test images have the same texture and color statistics). However, if the images are consistently color-shifted (for instance by changes in the spectrum of the light source) the performance will drop. If the network is able to cope with the color-shift with a negligible drop in performance one can say that the network is insensitive (or tolerant) to that color-shift. Setting an arbitrary threshold on the network performance one may define a *tolerance region* in the color space so that performance drops less than this value. This tolerance region is a description of the insensitivity of the network to color shifts, similarly to the MacAdam ellipses for humans. Obviously, tolerance regions in humans (as classically defined) and in machines (as defined here) are not identical concepts, but a convenient analogy to compare their behaviours.

*Will these (artificial) tolerance regions have something in common with the human insensitivity (MacAdam ellipse) region around the white?* Alignment between these two concepts would suggest a common explanation of both behaviours.

In order to check the above in different training environments one must: (1) train the considered networks for the task in the different environments and (2) test the tolerance of those networks in scenes where color has changed in a consistent form (that can be systematically represented in the chromatic diagram).

In the next subsections we discuss how to introduce systematic color changes in photographic images via simulated changes in spectral illumination and how this can be used to illustrate human color discrimination around the white.

### 2.3 Systematic color-shifts via changes in spectral illumination

To test the tolerance of the segmentation networks to color shifts in a meaningful way, one should use convenient ways to generate systematic, chromatically-controlled and consistent changes in the images of the different environments so that the networks face new (equivalent and controlled) situations not considered in the training.

The required color shifts in the test sets can be introduced in different ways. In the context of color constancy, different approaches have been used to model color changes in the images. These different approaches represent different degrees of approximation to the physics of image generation. The approximations differ on how well the geometry of rendering and the spectrum-to-tristimulus transforms are taken into account. Approaches to include consistent color-shifts which are progressively closer to the physics of image generation include:

1. Following simple models of illumination compensation (Finlayson et al., 1993; Chong et al., 2007), one should express the color of the images in certain tristimulus space and introduce independent linear variation in the tristimulus values. This is clearly better than naive operation in RGB digital counts, but the diagonal linear transform is still rather restrictive: the authors recommended this when the intrinsic dimensionality of spectral reflectance of surfaces and spectral radiance of the illuminant is as low as two or three.
2. Following general linear models of illumination compensation (Webster and Mollon, 1997; Clifford et al., 2007), one could apply a rotation and a scaling matrix to the tristimulus values. This transform is more general than the previous method based on diagonal matrices but still disregards the huge dimensionality reduction process that happens in the spectrum-to-tristimulus transform.
3. Virtual environments [such as CARLA simulator (Dosovitskiy et al., 2017)] are appealing to change the chromaticity of the illumination because they consider the 3-dimensional scene in the rendering. However, conventional programs usually make gross approximations from the colorimetry point of view: spectral distributions are not controllable and they usually operate in RGB digital counts. As a result, it is not obvious how to control the changes in the illumination to systematically sample chromatic directions to check discrimination in the 1931 CIE xy diagram.
4. A convenient alternative is assuming that the original images come from certain spectral reflectances under a given spectral illumination and recreating new images by applying the tristimulus equation assuming Lambertian surfaces with no mutual illumination. As opposed to methods that operate on tristimulus values, this method does take into account the huge dimensionality reduction in the spectrum-to-tristimulus transform, so illumination change is richer than a rotation+scaling in the tristimulus space. However, this method has also been criticized because it disregards the nonlinearities that come from mutual illumination (Laparra et al., 2012; Deeb et al., 2018).
5. Create annotated scenes for segmentation using the unconventional virtual reality tools that take into account both the geometry and the spectral content of light and reflectance of surfaces, as for instance (Heasly et al., 2014).
6. Take real scenes where the spectral illumination can be physically modified and measure (take pictures) using colorimetrically calibrated cameras (Laparra et al., 2012; Gutmann et al., 2014), or spectro-radiometrically calibrated cameras (Foster et al., 2016; Nascimento et al., 2016).

Of course, the best methods (5th and 6th) are not straight forward. The 6th case implies building a database from scratch (in case of having the expensive measurement equipment). Moreover the mentioned databases that include physical changes in the spectra are not good for our purposes because the spectral change is uncontrolled or does not properly sample the chromatic diagram. Moreover, they are not annotated for segmentation. In the 5th case, one would have to build virtual scenes from scratch and then use the internal (non-standard) code for the objects to derive the image segmentation maps. Therefore, methods 5 and 6 are too complicated for the illustrative test sets that we want to generate to check the invariance/tolerance of the segmentation networks. Then, between the next two methods (3rd and 4th, each with advantages and shortcomings) we chose the 4th method for its balance between complexity and colorimetric realism.

### 2.4 Human color discrimination illustrated via changes in the spectra

After the previous discussion about the different ways to introduce the color shift, here we describe in more detail the chosen option. Particularly, here we describe the change of tristimulus values of a surface of known spectral reflectance when we change the spectral illumination, and then we explicitly illustrate how uniform changes in hue and saturation over the chromatic diagram are not perceived uniformly. This anisotropic tolerance to color shifts,^4^ known as the MacAdam ellipses (Wyszecki and Stiles, 2000; MacAdam, 1942), is the human behaviour that we want to compare with the invariance region of the models.

Given an object of spectral reflectance, ρ_λ_∈[0, 1], illuminated by an illuminant with spectral radiance *s*_λ_ in *W*/*m*^2^*str*, its tristimulus values in certain color representation, *T*_*i*_ with *i* = 1, 2, 3, are given by Wyszecki and Stiles (2000):


(1) $$\begin{array}{l} T_{i}=k_{m}\int_{380}^{770}\rho_{\lambda }s_{\lambda }{\bar{T}}_{i}\left(\lambda \right)d\lambda \end{array}$$

where ${\bar{T}}_{i}\left(\lambda \right)$ are the *color matching functions*, or the sensitivity of the color sensors in that representation, and *k*_*m*_ = 683*lm*/*W*, is the *luminous efficacy* constant. This implies that the *chromatic coordinates*, $t_{i}=T_{i}/\underset{i}{\sum }T_{i}$, also change with the illuminant.

**Figure 2:**
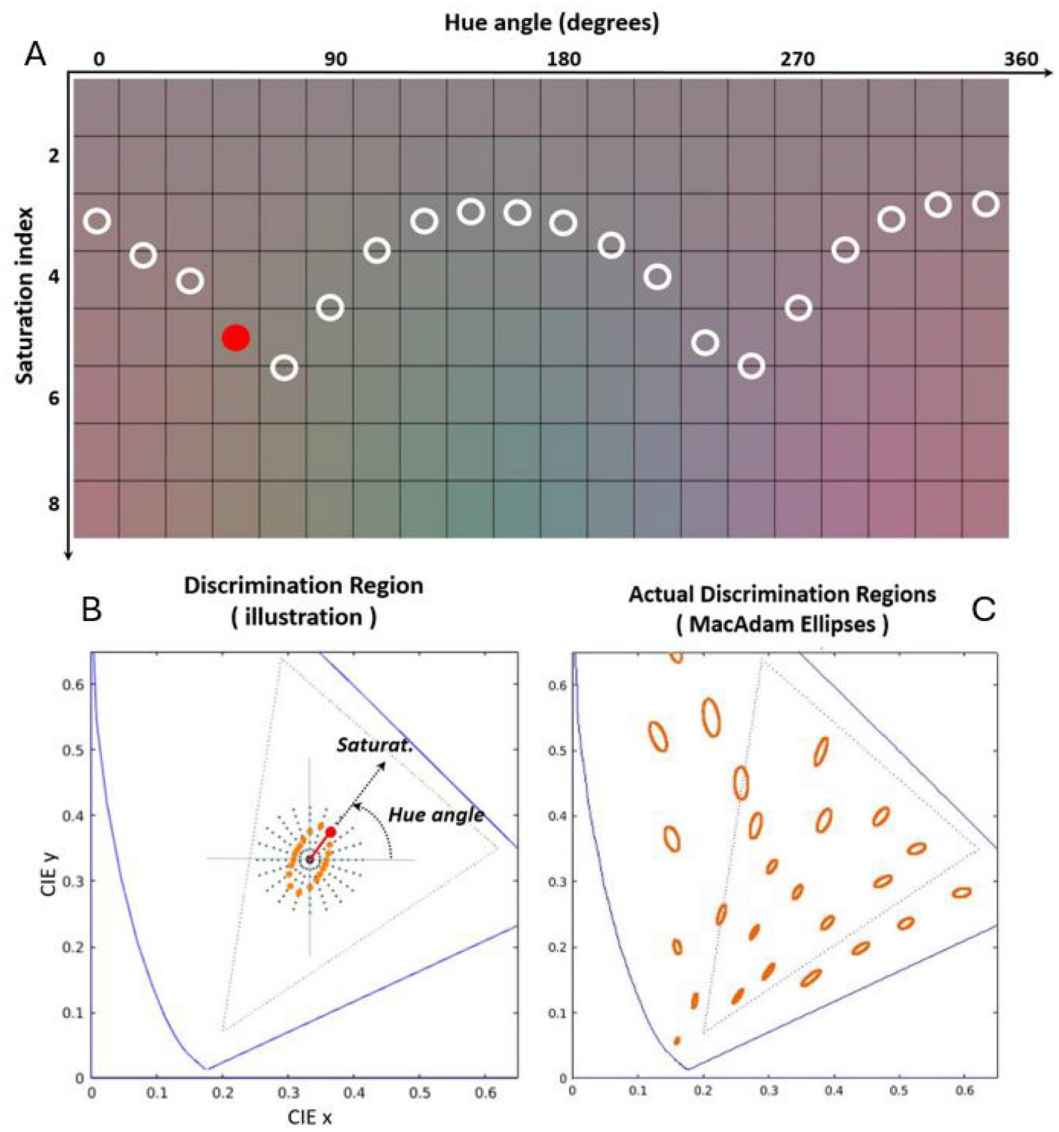
Illustration of human color discrimination: tolerance to saturation for different hues. **(A)** Shows patches of flat spectral reflectance illuminated by sources with spectral radiances selected to cover the 1931 CIE xy diagram as seen on a standard CRT display (Malo and Luque, 2002). Black dots in the CIE xy chromatic diagram of **(B)** show the polar distribution of the chromaticity of the considered illuminants. The illuminants are organized as a function of hue and saturation, i.e. angle with respect to the *x* axis, and distance with respect to the white point respectively. For each hue [each column in the colored panel of **(A)**] the Euclidean distance in the chromatic diagram required to induce certain perceptual departure from the white color of the same luminance is different. That is why the insensitivity region around the white [determined by the circles in **(A)**] is an ellipse with certain orientation [orange dots in **(B)**]. The diagram in **(C)** displays the insensitivity regions for humans measured by MacAdam (1942) at a number of color locations over the chromatic diagram.

Figure 2 shows the variation of the color appearance of a flat reflectance, ρ_λ_ = 1∀λ, when it is illuminated by a set of sources with spectral radiances, $s_{\lambda }^{⋆}$, taken so that the color of the sample has the desired tristimulus vectors, *T*^⋆^, with chromatic coordinates represented in the 1931 CIE xy diagram at the left and a constant luminance of 35 *cd*/*m*^2^. The spectral sources were computed via:


(2) $$\begin{array}{l} s_{\lambda }^{⋆}=\underset{s_{\lambda }}{arg min}|T^{⋆}-T\left(s_{\lambda }\right){|}_{2} \end{array}$$

where *T*(*s*_λ_) was computed as in Equation 1. Metamerism means that Equation 2 is ill-posed (Wyszecki and Stiles, 2000). The algorithm we use^5^ breaks the multiplicity of solutions by looking for the illuminant that minimizes the error in tristimulus values using an exhaustive search in a structured dataset of 20,000 spectral radiances/reflectances. The structure of this dataset (the way the spectral shapes are ordered) is based on the Munsell book of color. This guarantees that the considered spectra represent a perceptually uniform sampling of the color space. In this example the considered illuminants are organized as a function of *hue* and *saturation*, i.e., angle with respect to the *x* axis, and distance with respect to the central white point respectively.

The uniform distribution of color variations in a polar representation along the 1931 CIE xy diagram in Figure 2A illustrates the fact that that human color discrimination is not isotropic around the white, i.e., it is non-uniform. Note that when linearly increasing the saturation of the color along the different hue directions (going down along each column of the colored panel), the perception of *colorfulness* (Fairchild, 2013) is not uniform. See that, qualitatively and just for illustrative visualization, the circles in the colored panel define a boundary between clearly chromatic patches (below the curve) and mainly achromatic patches (above the curve). This human region of tolerance or invariance around the white can be plotted in the chromatic diagram (ellipse represented by the orange dots in Figure 2B) by using the corresponding cartesian to polar transform. As an example of this transformation, see for instance the position of the solid circle located in the colored panel Figure 2A (fourth hue and fifth saturation index) and its corresponding location in the chromatic diagram.

Figure 2 is just a compelling visual illustration of the anisotropy of human tolerance to color shifts: the tolerance is maximal in the *yellow-blue* direction and minimal in the *red-green* direction. Of course, this visualization is not an accurate measurement of the color discrimination ellipse (MacAdam, 1942; Jennings and Barbur, 2010; Alabau-Bosque et al., 2024). Interestingly, even though this visualization has all the limitations of color reproduction in displays (Hunt, 2005), the anisotropy of human tolerance to color shifts is so robust that the characteristic two-minima-shape of the achromatic-to-chromatic boundary is clearly visible. Note that the orientation of this qualitatively drawn boundary-and-ellipse is consistent with the classical experimental ellipses (MacAdam, 1942; Wyszecki and Stiles, 2000) depicted in Figure 2C.

We will be back to this two-minima shape in the hue-saturation plane and the associated ellipsoid when we present the results of the tolerance regions of the segmentation networks in Section 3.

### 2.5 Spectral illumination changes in the environments

Once the networks are trained in the considered environments, the scenes are modified to introduce changes in spectral illumination using the sources, $s_{\lambda }^{⋆}$, shown in Figure 2. To do so, spectral reflectances have to be associated to each region of the original scenes. This association is done by assuming that the tristimulus vectors, *T*, in the original scenes (e.g., the chromaticities in Figure 1 with their corresponding luminances) come from the illumination of certain reflectances, $\rho_{\lambda }^{⋆}$, with an equienergetic illuminant:


(3) $$\begin{array}{l} \rho_{\lambda }^{⋆}=\underset{\rho_{\lambda }}{arg min}|T-k_{m}\int_{380}^{770}\rho_{\lambda }{\bar{T}}_{i}\left(\lambda \right)d\lambda {|}_{2} \end{array}$$

**Figure 3:**
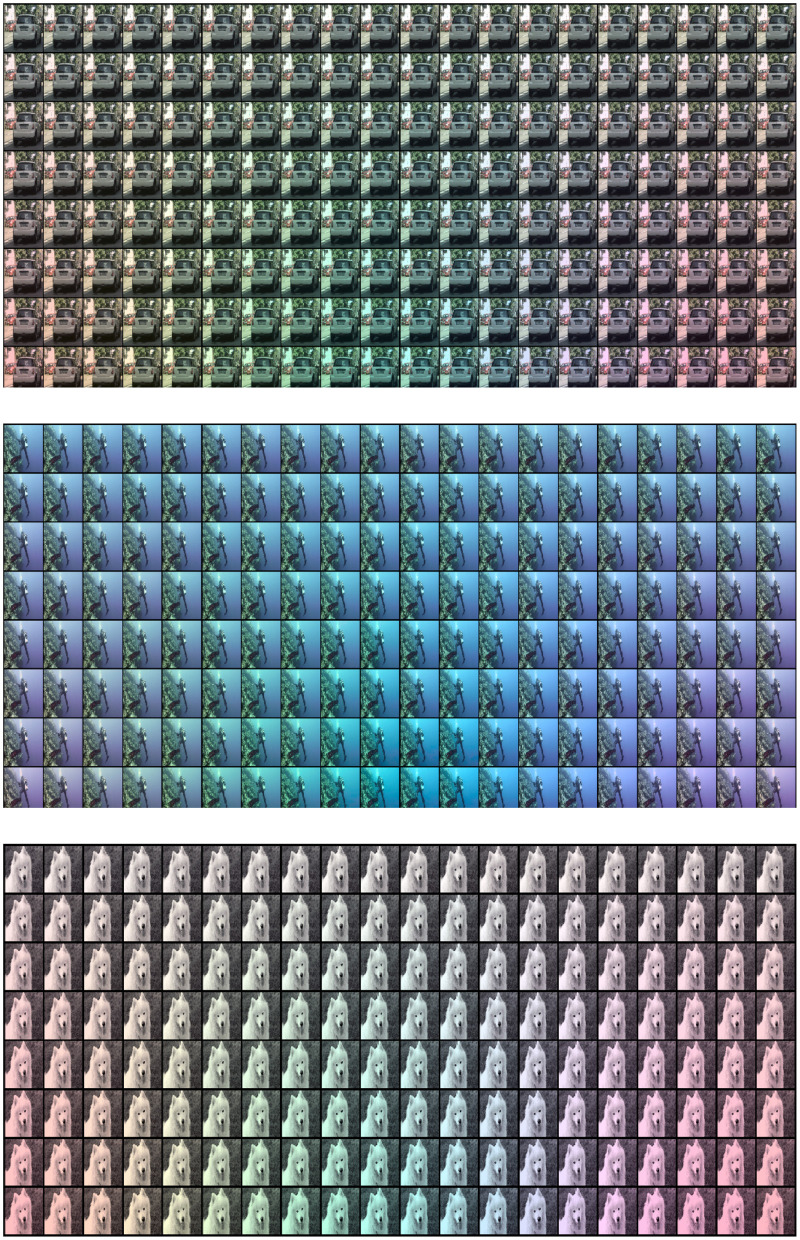
Scenes with modified illumination starting from a different original image: natural **(top)**, underwater **(center)**, and flat-reflectance, i.e., achromatic **(bottom)**.

Again, the solution of the ill-posed Equation 3 was obtained through the function tri2spec.m of Colorlab (Malo and Luque, 2002) because the spectra in the Munsell database do a thorough sampling of the color space. Once each pixel has an associated reflectance, $\rho_{\lambda }^{⋆}$, its new color, *T*′, under the new illumination is computed with Equation 1 using $s_{\lambda }^{⋆}$. Finally, the new 1931 CIE XYZ colors are transformed into digital values assuming a standard display calibration (Hunt, 2005; Malo and Luque, 2002). Figure 3 shows an example of the result of this procedure applied to one image of each of the three different training environments considered.

The saturation of the considered spectral sources was limited by the fact that we did not want the manifold of modified colors to lie outside the triangle of primary colors in a regular display.

These modified scenes can be used to test each of the image segmentation networks which were trained on the three different original scenes. The performance is expected to be similar for illuminants with small spectral contrast: the segmentation results for the scenes of the first row in Figure 3 will be similar to the performance on the original scenes. However, it is expected to change for illuminants of bigger saturation and different hues (down along the different columns).

### 2.6 Networks for image segmentation

**Figure 4:**
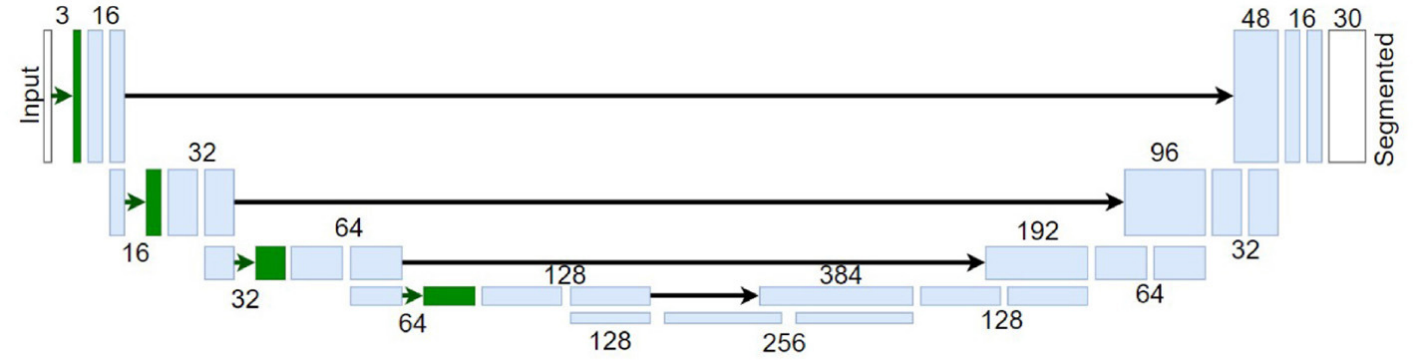
Illustrative U-Net architecture for image segmentation. The blocks in *blue* represent regular convolutional layers, and the blocks in *green* represent bio-inspired *Divisive Normalization* layers. Numbers by the layers indicate the number of features and black arrows represent the skip unions.

In this work, we used U-Nets networks to perform image semantic segmentation following the state-of-the-art for this visual task (Ronneberger et al., 2015). See Figure 4 for an illustration of this architecture. In these networks, the input images (in digital values) go through a set of layers with progressively lower spatial resolution, i.e., the image dimensions decrease as the image passes through each block. Also, each block has a progressively higher number of features, i.e., different attributes detected by the network, such as different patterns, to capture more complex information up to the network bottleneck. From this inner representation, the signal is spatially expanded again up to the original resolution ending with a layer (in white in the figure) with a number of features equal to the number of distinct classes to be identified. Part of the high-resolution information is passed from the early layers to the late layers after the bottleneck through the so-called skip connections. The final layer performs the classification of each pixel to one of the possible classes in the dataset, i.e., assigned to the class that achieves higher response in this final layer. Note that this implies that this layer depends on the number of possible classes of the considered dataset and therefore a model trained in one dataset can not be applied to a different dataset with a different number of classes.

Apart from the standard U-nets, we considered the biologically inspired modification proposed in Hernández-Cámara et al. (2023b). This modified architecture considers *Divisive Normalization* (DN) layers in the encoding part of the U-Net (layers depicted in green in Figure 4). This nonlinear computation, y=N(x), is relevant because the response of each unit, *x*_*i*_, is normalized by a pool of the responses of the neurons tuned to neighbour features:


(4) $$\begin{array}{l} y_{i}=sign\left(x_{i}\right)\frac{\left|x_{i}\right|}{b_{i}+\sum_{j}H_{ij}|x_{j}|} \end{array}$$

and this normalization has proven to be important to explain both chromatic adaptation (Abrams et al., 2007; Hillis and Brainard, 2005; Fairchild, 2013) and contrast and texture adaptation (Watson and Solomon, 1997; Martinez et al., 2019). This previous literature on the benefits of Divisive Normalization for adaptation suggests that U-Nets with Divisive Normalization may be more tolerant to changes in illumination, and their insensitivity regions may be more similar to those of humans.

## 3 Experiments and results

In this section, the considered networks are first trained and evaluated for the image semantic segmentation task on the different environments. Then, the models are tested in the scenes under the new spectral illuminations covering a range of hue and saturation values to see the shape of the tolerance region to changes in illumination. We show that in the naturally illuminated environment human-like tolerance regions emerge, but they do not in the counter-example environments where the color statistics are markedly different.

### 3.1 Model training and segmentation performance

In both kinds of architectures (without and with Divisive Normalization) the parameters of the nets are obtained via supervised learning: the models are trained to minimize a measure of the segmentation error over a set of images from the considered original environments. In this case, the selected measure was the Mean Absolute Error (MAE), which is maximised if, for each pixel, the correct class is predicted with probability one and the other classes have probability zero, and therefore lower MAE is better. The final performance of the networks was measured using the Intersection over Union (IoU) measure (Rahman and Wang, 2016) over the validation data, a subset of the 20% of the training images that are not used in the training process. IoU takes into account the predicted area and the real area for each class and how much they intersect and therefore higher is better, with IoU ∈[0, 1] . We train each network during 200 epochs (each complete pass of the whole training data) using Adam as the optimizer (Kingma and Ba, 2014) and a batch size of 16 images. We keep the model parameters that achieve higher IoU on the validation data, which we compute after each epoch.

We trained *six* artificial systems performing image semantic segmentation: 2 *architectures* × 3 *environments*. This includes *two* biologically interesting cases (both architectures trained on the naturally illuminated images under daylight source), and *four* counter-examples: the ones trained in environments with non-natural illumination (underwater) or spectrally flat reflectances (achromatic images).

Given that the encoding part of the considered networks has certain resemblances with the retina-cortex pathway (Jacob et al., 2021), and the aforementioned biological inspiration of the divisive normalization layer (Abrams et al., 2007; Hillis and Brainard, 2005), our U-nets *have the ability* to use color information to solve segmentation. However, as other features (e.g., edges, shape, and textures) may also contribute to the solution of the problem there is no guarantee that these nets develop human-like tolerance to color shifts. The counter-example case that consists of achromatic images particularly was chosen to ensure that the networks trained in this condition do not use color information at all.

We tested the performance of the considered nets (U-Net and U-Net+DN) in the three environments where they were trained (numbers in bold-face in Table 1). Moreover, we did two extra tests in order to check the relevance of color in the segmentation problem. To do so we considered the databases that originally consisted on scenes under natural daylight illumination (CityScapes and Pets). In particular, we removed the color information in CityScapes, and we recovered the original color information in Pets (numbers in light-face in Table 1).

**Table 1:** Segmentation performance: test IoU results (mean ± standard deviation) of the models trained in the different environments when performing 300 evaluations over subsets of the test images.

**Training env**.	**Color Natural Illum**.		**Underwater Illum**.	**Achrom. images**	
**Test env**.	**Color**	**Achrom**.	**Color**	**Achrom**.	**Color**
U-Net	**0.77**±0.02	0.54 ± 0.03	**0.66**±0.05	**0.77**±0.02	0.72 ± 0.04
U-Net + DN	**0.78**±0.02	0.68 ± 0.02	**0.70**±0.04	**0.80**±0.02	0.66 ± 0.04

To test each model we perform 300 realizations where we randomly select 20 test images from their corresponding test set and compute the IoU performance. Table 1 summarizes the results.

First, as expected results show that using the model with DN layers generally improves the segmentation results (compare first and second row of numbers in bold). Second, when comparing the color segmentation importance, the most important factor seems to be consistency with training, i.e., the models trained with color images get worse when removing color information, and the models trained with achromatic images get worse when facing color images (compare columns in bold and light in the non-underwater environments). However, if we compare the reductions in performance, we see higher reductions in the color-trained models tested with achromatic images (21% average reduction in IoU) than in the achromatic-trained models tested with color images (12% average reduction in IoU). This highlights that color is certainly beneficial for segmentation.

In order to check the significance of the differences between the performances seen in Table 1, we carried out a Mann-Whitney U-test (Mann and Whitney, 1947). In this non-parametric test the null hypothesis is that the distribution of the set of samples of a variable is the same as the distribution of the samples of another variable. Therefore, rejection of the null hypothesis implies that the compared variables are significantly different, i.e. one is larger than the other (Howell, 2013; Corder and Foreman, 2009). Table 2 shows the U-statistic over the number of samples (the effect size, or the proportion of pairs that support that items from group 2 are larger than items from group 1) and the corresponding p-values for all the different comparisons. We compared the IoU performance of the no-DN vs the DN models within each training environment (Table 2 top). We also compared the performance in the chromatic vs the achromatic version of the datasets (Table 2-bottom). In all the cases the null hypothesis was rejected (all *p*-values < 0.001), meaning that all the differences are significant. It is important to note that comparisons can be made only within each training environment because, as stated in the model definition, each dataset has a different number of classes and intrinsic difficulty.

**Table 2:** Significance of the differences in segmentation performance: Mann-Whitney U-test statistic and *p*-values for the different comparisons: models (top), and achrom/color tests (bottom).

**Training env**.	**Color natural illum**.		**Underwater illum**.	**Achrom. images**	
**Test env**.	**Color**	**Achro**	**Color**	**Achro**	**Color**
MW-stat (no-DN vs. DN)	0.70	0.9998	0.73	0.76	0.82
p-val (no-DN vs. DN)	1.1·10^−16^	1.2·10^−99^	1.7·10^−22^	4.4·10^−29^	5.9·10^−43^
**Training env**.	**Natural Illum**.		**Achro illum**.	
**Model**	**U-Net**	**U-Net+DN**	**U-Net**	**U-Net+DN**
MW-stat (Achrom. vs. Color)	1.0	0.9998	0.90	0.99
p-val (Achrom. vs. Color)	1.1·10^−99^	1.3·10^−99^	1.1·10^−65^	2.9·10^−99^

### 3.2 Tolerance to illuminant change in segmentation networks

To test the tolerance to illuminant changes we evaluate the segmentation performance of the different networks (trained with the three different types of images and the two types of architecture) with the color-shifted scenes. We do the evaluation 300 times with subsets of the test images, following the same procedure we did to obtain the results in Table 1. We compare the performance of the models with images with spectral changes along the hue-saturation plane with regard to their results on their training set from Table 1. Then, we can define the tolerance/invariance region of a model as the hue and saturation combinations where the results change less than a certain threshold.

**Figure 5:**
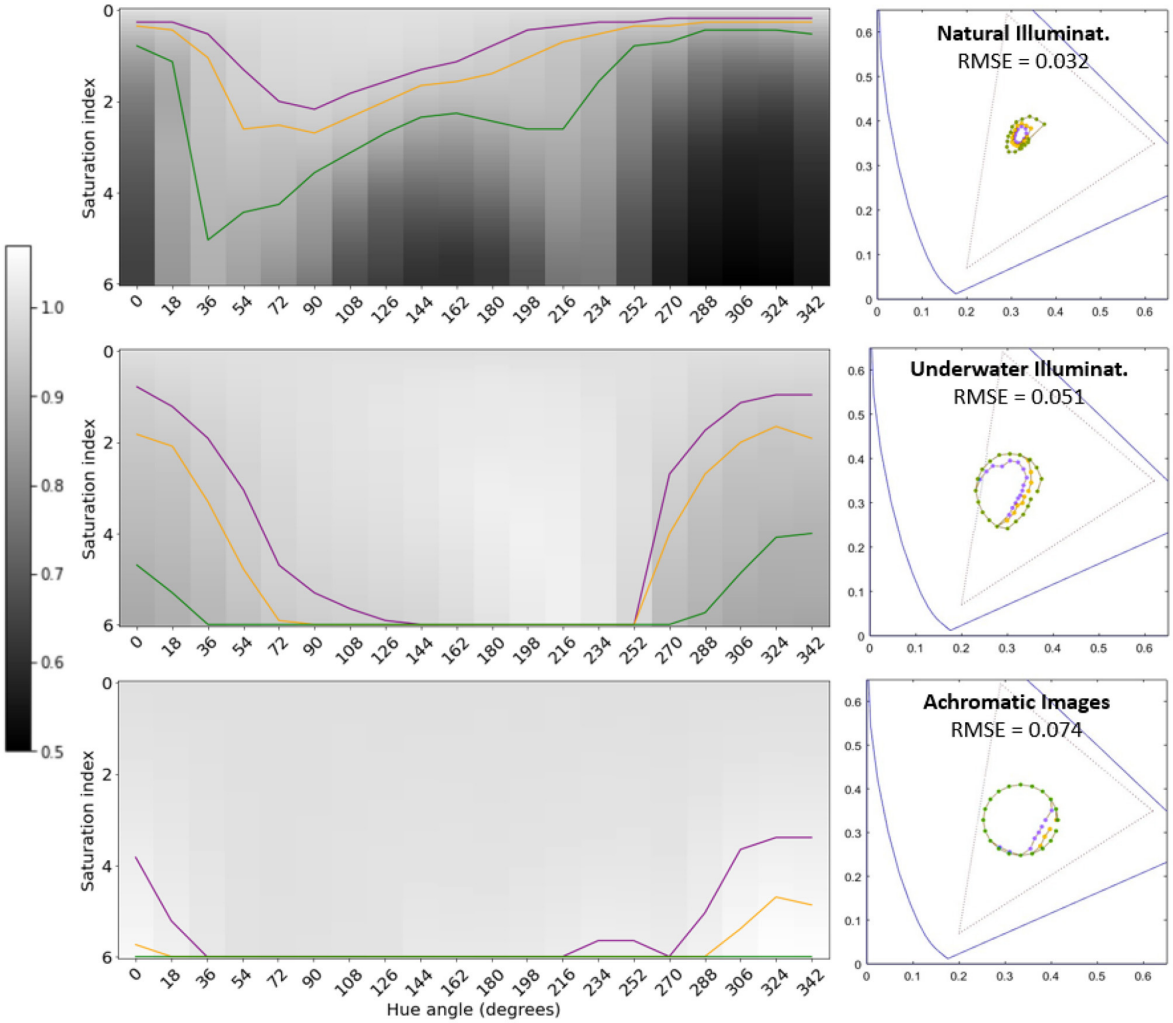
Tolerance of segmentation performance to illuminant change for different environments (regular U-Nets). The results of the *natural, underwater*, and *achromatic* environments are represented in the **top**, **middle**, and **bottom** rows respectively. Gray level represents the segmentation performance under different illuminations with regard to the reference performance obtained for the original scenes. Darker values represent lower performance. The curves in *purple, orange* and *green*, represent variations of the performance of 3%, 5%, and 10%, respectively. These curves define *tolerance regions* for performance in the chromatic diagram. The RMSE values represent the distance between the average of these tolerance regions in the artificial system and the corresponding tolerance ellipse in humans.

**Figure 6:**
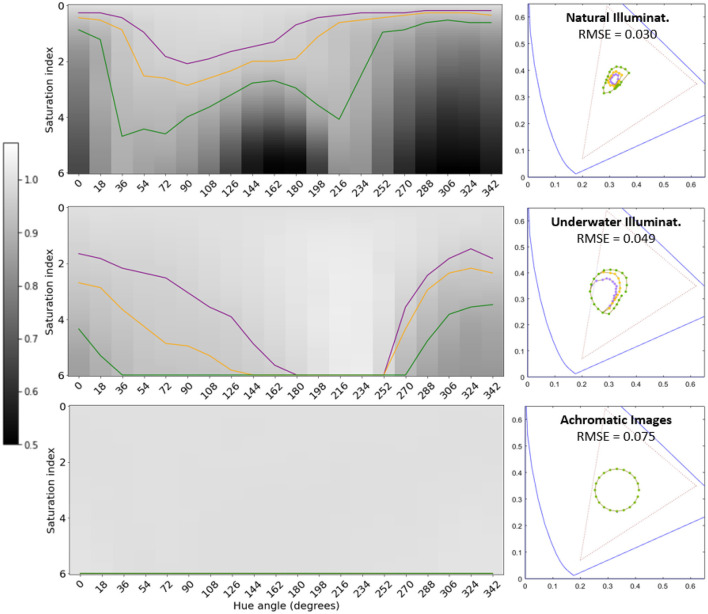
Tolerance of segmentation performance to illuminant change for different environments (U-Nets with Divisive Normalization). Same results as in Figure 5, but for the architecture with Divisive Normalization.

Figures 5, 6 show the variation of the segmentation performance as a function of the change of *saturation* and *hue* of the illuminant for the different architectures and the different environments considered. It also shows the corresponding *tolerance regions* for 3%, 5%, and 10% changes in performance with regard to the training situation. Finally, Figures 5, 6 also show the corresponding regions in the 1931 CIE xy diagram.

The gray level in the first row (zero saturation) of the *saturation-hue* planes represents the IoU performance of the segmentation network in the original scenes. The values of Table 1 are taken as reference in each case. Then, darker or lighter values for other illuminants correspond to lower or higher performance in the image segmentation task with regard to its reference. In particular, the curves in *purple, orange*, and *green*, represent variations of the performance of 3%, 5%, and 10%, respectively, with regard to their reference. Therefore, these curves also represent *tolerance regions* in the chromatic diagram where the performance departs from the original reference less than a certain threshold.

The specific size of the tolerance regions of course depends on the (arbitrarily) selected threshold for the departure with respect to the reference value. However, the fact that, given a threshold, the scale of the region is fundamentally different for the different environments is certainly relevant. Moreover, the (non-circular) shape and orientation of the regions indicate that the segmentation function learnt in a certain environment may imply anisotropies of the robustness of the (artificial) visual system under changes of illumination.

Chromatic diagrams in Figures 5, 6 display the root mean square error (RMSE) distance (in chromatic coordinates) between the mean 3% tolerance regions over the 300 iterations in the artificial systems and the corresponding color discrimination MacAdam ellipse in humans. For this comparison, the tolerance region in humans for that specific chromatic location was obtained by interpolating the parameters of the three closer ellipses out of the 25 regions measured in MacAdam (1942). In the corresponding (human and artificial) regions we took 20 points at uniformly distributed angles and we computed the average distance between the corresponding points at those angles, leading to the reported RMSE value (in chromatic coordinates).

Results show an interesting alignment of the anisotropy of artificial systems with human anisotropy but only for natural scenes under daylight illumination. The counter-examples with unusual color statistics lead to non-human tolerance regions and anisotropies. In both counter-example environments, the performance is more insensitive to the changes in illumination, and this is particularly true for the architectures trained on images with flat spectral reflectance (achromatic images). As a result, the tolerance regions are substantially bigger for the same thresholds, and the insensitivity is more isotropic.

There may be two causes for this effect. On the one hand, the underwater scenes seem to have a wider color gamut with a smaller peak in the probability of colors around the mean (see scatter plots in the diagrams of Figure 1 and the corresponding ellipses representing the covariance matrices). This wider spread of colors (wider than in scenes in daylight illumination) would explain the bigger tolerance to color change of the systems trained in this unusual environment. On the other hand, the segmentation systems trained on images of flat spectral radiance may be insensitive to color just because (by construction of the training set) their ability for segmentation has to be based on non-chromatic features. Therefore, substantial changes of color should not affect much their performance, leading to big (and isotropic) tolerance regions.

The effect of the considered architectures in the size and orientation of the sensitivity regions is secondary: although the absolute performance of the networks equipped with Divisive Normalization is better (see Table 1 and slightly bigger areas of the insensitivity regions), this has low impact on the anisotropy depending on saturation and hue. The differences in the shape of the tolerance regions depend more strongly on the different image statistics rather than on the considered architectures.

**Figure 7:**
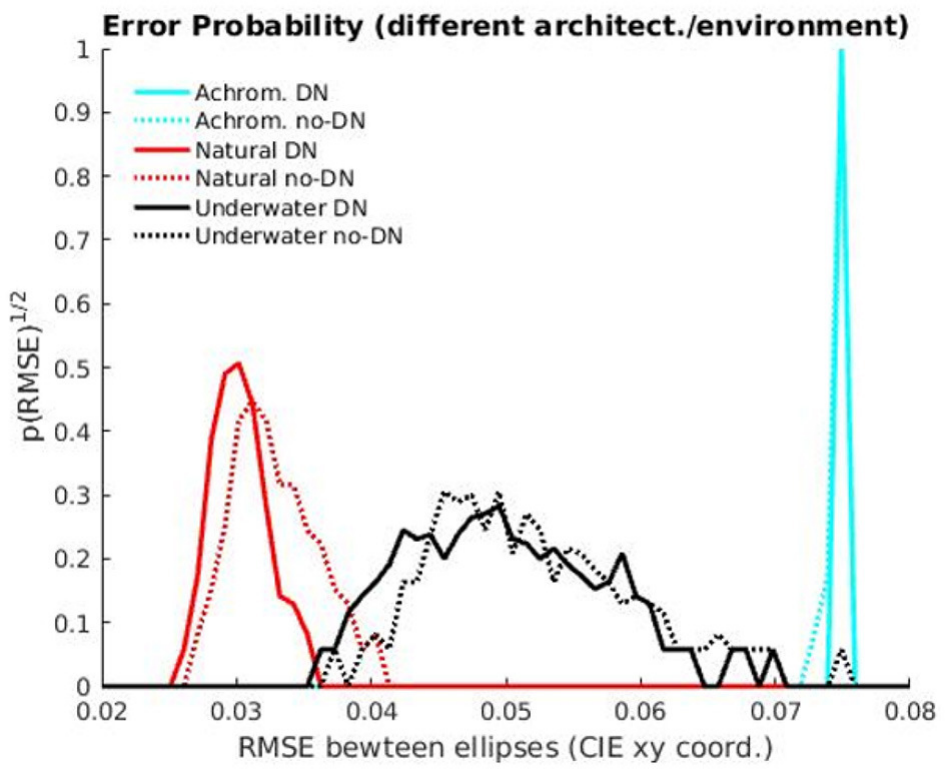
Distances to MacAdam ellipses: Histograms of the RMSE errors comparing the human MacAdam ellipses and the tolerance region of the models for 300 realizations with test subsets.

To confirm the statistical significance of the results mentioned above we display the distributions of errors with the human discrimination ellipse and we perform non-parametric Kolmogorov-Smirnov tests to check if these samples of errors come from the same distribution or not. Figure 7 shows the histograms of the RMSE between the tolerance region of the models and the human MacAdam ellipse for the 300 realizations performed in the evaluation.

The distances between the histograms of errors confirm that the environment is the major factor in getting human-like ellipses, and it is way more important than the explored variants of the architecture. Of course, the 2-sample non-parametric Kolmogorov-Smirnov tests also confirm that these big differences (basically non-overlapping histograms for the different environments) are significant, with *p* < 0.001, (see the test statistics and the *p*-vaues in Table 3). The results of different architectures only introduce slight shifts in the histograms, so this is clearly a secondary (less relevant factor). In fact, the KS-tests reveal that differences in architecture are not significant in the non-natural cases (underwater images and achromatic images, in black and cyan, *p* > 0.001), but they are for the natural images (in red, *p* < 0.001). The histograms reveal that the significance of the difference between no-DN and DN networks according to the KS-test for natural images does not modify the fact that the environment is way more important than the architecture to get human-like results. Interestingly, the significance of the difference between the errors in the DN vs. no-DN case for natural images means that in the ecologically sensible situation, DN is important to increase alignment with humans, as expected from the rationale suggested in Hernández-Cámara et al. (2023b, 2024).

**Table 3:** Significance of the differences of RMSE errors.

		**Natural illum**.		**Underwater**		**Achromatic**	
		**No DN**	**DN**	**No DN**	**DN**	**No DN**	**DN**
Natural illum.	No DN	-	0.79	0.9998	0.9992	1.0	1.0
	DN		-	1.0	1.0	1.0	1.0
Underwater	No DN			-	0.56	0.998	0.998
	DN				-	1.0	1.0
Achromatic	No DN					-	0.52
	DN						-
		**Natural illum**.		**Underwater**		**Achromatic**	
		**No DN**	**DN**	**No DN**	**DN**	**No DN**	**DN**
Natural Illum.	No DN	-	4.2·10^−35^	1.3·10^−99^	2.2·10^−99^	1.9·10^−111^	1.1·10^−113^
	DN		-	1.1·10^−99^	1.1·10^−99^	1.9·10^−111^	1.9·10^−113^
Underwater	No DN			-	0.01	8.2·10^−111^	4.4·10^−113^
	DN				-	1.9·10^−111^	1.1·10^−113^
Achromatic	No DN					-	8.9·10^−5^
	DN						-

## 4 Discussion and conclusions

### 4.1 Summary of results

Artificial networks trained for image segmentation develop human-like tolerance to changes in illumination (around the white) when they are trained on natural images under daylight illumination. Similarly to humans, these networks are more tolerant to variations in the yellow-blue direction rather than in the red-green direction: see the similarity between two-minima the curve in the colored saturation-hue panel of Figure 2 and the shape of the performance surfaces in the top panels of Figures 5, 6. This anisotropy occurs both for regular U-Net architectures, Figure 5-*top row*, and with architectures augmented with the biologically-inspired Divisive Normalization, Figure 6-*top row*.

However, alternative environments with markedly different image statistics (e.g., underwater scenes and achromatic scenes) lead to systems in which the tolerance to color changes is not aligned with human color discrimination (substantially bigger insensitivity with lower anisotropy), Figures 5, 6-*middle and bottom rows*.

### 4.2 Function, architecture, or just image statistics?

The reported emergence of a human-like anisotropy in the tolerance to color changes in artificial systems trained in a natural environment with natural illumination means that image segmentation (which is, at least partially, based on color) *could be* the principle behind the development of the anisotropy observed in humans for color discrimination.

However, not all the explanations can be attributed to the specific *segmentation* function. First, lower-level functions that involve local equalization of the color manifold, such as *error minimization* and *information maximization* (Laparra et al., 2012; Laparra and Malo, 2015; da Fonseca and Samengo, 2016, 2018), also lead to this kind of asymmetry. See that the ellipses from the local-PCA in Figure 1-*left and middle* (good for local equalization) have qualitatively similar properties as the tolerance regions that emerge in the segmentation networks. Second, more than the function, it is the data distribution that may lead to the observed asymmetry in the behaviour of the networks. In fact, statistical analysis (Figure 7 and associated Kolmogorov-Smirnov tests) shows that a natural color distribution is the major factor in getting human-like ellipses. The following example connects a strong physical constraint with a major asymmetry in the color data that may explain differences in performance and discrimination. If the spectrum of the sunlight at different times of the day can be approximated by a black-body radiator (Malo and Jiménez, 2011; Jiménez and Malo, 2014) the manifold of natural colors will be elongated along the Planckian locus in the 1931 CIE xy diagram. This locus, for the white (Wyszecki and Stiles, 2000), approximately has the orientation of the ellipse in Figure 1-*left*, and the regions of Figures 5, 6 *Top*. This makes sense because the natural dataset will have multiple examples of similar objects with different illuminations along that (yellow-blue) direction. As a result, in order to obtain good segmentation performance, the networks (of whatever architecture) have to be more invariant to changes of illumination in that direction. Third, the counter-examples of markedly different color statistics imply that the same functional goal leads to very different tolerance regions.

Finally, the architecture selected to perform the image segmentation does not seem to have a big impact on the alignment of the asymmetries of humans and networks (see histograms in Figure 7). In fact, the functions related to information maximization and error minimization reduce to local PCA (Laparra et al., 2012), and hence they are independent of the architecture. In Figure 1 we see that local PCA leads to regions which are similar to the tolerance regions found in the different image segmentation networks when trained in similar environments, see Figures 5, 6. However, there is a small, but statistically significant difference (histograms in red in Figure 7 with hypothesis-zero rejected by KS-test with *p* < 0.001) that suggests that the Divisive Normalization is important to improve the alignment with humans in color discrimination in the ecologically significant case. This is consistent with the suggestions done in Hernández-Cámara et al. (2023b, 2024).

### 4.3 Conclusions

Artificial networks for image segmentation trained in natural environments with natural illumination exhibit human-like tolerance to changes in illuminant, aligning with human color discrimination. This is the first report on the emergence of the alignment of image segmentation networks with human color discrimination. However, in environments with markedly different image statistics, the tolerance to color changes in these artificial systems deviates from human color discrimination. This suggests that the regularities of the environment are much more significant in shaping the behaviour for color discrimination than the architecture of the image segmentation network. This is in contrast with other chromatic properties, e.g., color induction (Gomez-Villa et al., 2020) or color CSFs (Li et al., 2022), where the architecture strongly modifies the human-machine similarities. In fact, in the discrimination case considered here, alternative functional principles such as error minimization or information maximization (Laparra et al., 2012; Laparra and Malo, 2015; da Fonseca and Samengo, 2016, 2018) which only depend on the data (e.g., local PCA), also lead to tolerance regions of human-like orientation if applied in the proper environment. In conclusion, the anisotropy in human color discrimination is also present in segmentation neural networks. This is probably due to the adaptation of (both natural and artificial) neural networks to the color data distribution.

## Data Availability

The datasets presented in this study can be found in online repositories. The names of the repository/repositories and accession number(s) can be found below: https://github.com/pablohc97/SegmentationModelsAlignedColorDiscrimination.

## Footnotes

^1^ https://www.cityscapes-dataset.com/

^2^ https://irvlab.cs.umn.edu/resources/suim-dataset

^3^ https://www.robots.ox.ac.uk/~vgg/data/pets/

^4^Here color-shifts modelled as changes in the spectral reflectance or spectral radiance.

^5^The function tri2spec.m of Colorlab (Malo and Luque, 2002).
